# Implications of the Relationship Between Refractive Error and Biometry in the Pathogenesis of Primary Angle Closure

**DOI:** 10.1167/iovs.62.10.38

**Published:** 2021-08-31

**Authors:** Kilhwan Shon, Kyung Rim Sung, Joo Young Yoon

**Affiliations:** 1 Department of Ophthalmology, Gangneung Asan Hospital, Gangneung, Korea; 2 Department of Ophthalmology, College of Medicine, University of Ulsan, Asan Medical Center, Seoul, Korea

**Keywords:** primary angle closure (PAC), refractive error, axial length (AL), ocular biometry, pathogenesis

## Abstract

**Purpose:**

The purpose of this study was to investigate the relationship between refractive error and ocular biometry and its implication in the pathogenesis of primary angle closure (PAC).

**Methods:**

We have retrospectively recruited 119 PAC eyes and 388 non-PAC eyes with an axial length (AL) of ≤25.0 mm and a spherical equivalent (SE) of ≥−6.0 diopters (D). Stepwise multiple regression was performed for keratometry value (K), AL, anterior chamber depth (ACD), and SE.

**Results:**

PAC eyes were more likely to be in women and have a higher IOP and shorter AL than non-PAC eyes. In a multiple regression analysis, SE was not associated with PAC. The associations between AL and SE or AL and ACD were not different in PAC eyes compared with non-PAC eyes. However, the cornea was flatter in PAC eyes (β = −0.448, *P* < 0.001), and a flatter cornea was associated with more hyperopic refraction (β = −0.454, *P* < 0.001) and shallower ACD (β = 0.073, *P* < 0.001) in PAC eyes. ACD was not associated with SE in non-PAC eyes, but shallower ACD was associated with greater myopic refraction in PAC eyes (*β* = 1.117, *P* = 0.006).

**Conclusions:**

PAC eyes seem to have flatter cornea compared with non-PAC eyes. A shallower ACD seems to be associated with greater myopic refraction in PAC eyes, but not in non-PAC eyes.

One of the features distinguishing primary angle-closure glaucoma (PACG) from primary open-angle glaucoma (POAG) is a shallow anterior chamber. This can be identified on a slit-lamp examination with the Van Herick technique or a gonioscopic examination.^1^ Anterior chamber depth (ACD) is the most widely measured parameter in the anterior chamber and is an essential part of the modern intraocular lens power calculation for cataract surgery.^2^ In addition, various parameters have been proposed for a more detailed measurement of anterior segment anatomic features, including the anterior chamber width, anterior chamber volume, angle opening distance, trabecular-iris angle, trabecular-iris space area, and trabecular-ciliary process distance, measured with imaging modalities like ultrasound biomicroscopy (UBM) or anterior segment optical coherence tomography (AS-OCT).^3^ Based on such measurements, several anatomic features have been suggested as contributing to primary angle closure (PAC) development, including a larger lens vault, larger iris volume, thicker iris, greater iris curvature, a plateau iris configuration, or anterior rotation of the ciliary processes.^4^^–^^13^ However, despite the importance of the refractive mechanism in eye function, only recently has a refractive error in patiens with PAC gained academic attention.

It has been consistently shown that both shorter axial length (AL) and hyperopia are not associated with shallower ACD in eyes with PAC, which is somewhat counterintuitive, as logic suggests that smaller eyes are expected to have more hyperopic refraction and shallower ACD.^14^^–^^18^ The logic is supported by evidence, such as that in the general population, a refractive error has been negatively associated with ACD, meaning that hyperopic eyes are expected to have a shallower anterior chamber.^19^^–^^21^ An even stronger correlation has been consistently demonstrated between ACD and AL in the general population in various ethnic groups. That is, longer eyes have a deeper anterior chamber – with the rare exception of extremely long eyes (AL ≥27.5 mm).^21^^–^^24^

We explored the relationship between four parameters – refractive error, corneal power (K), AL, ACD – and the presence of PAC with a cohort including both PAC and non-PAC eyes. We have broken down our objective into two questions: “is there a difference in the group average of four parameters between PAC and non-PAC eyes?” and “are the relationships among the four parameters different in PAC eyes compared to non-PAC eyes?” Given the correlation among four parameters and the complexity of these questions, we concluded that a single model would be insufficient. We have constructed separate stepwise multiple regression models for each of the four parameters and summarized the results accordingly.

## Methods

### Subjects

We retrospectively reviewed the electronic medical records of patients with PAC in the Asan Glaucoma Progression Study cohort between May of 2015 and April of 2018. Non-PAC subjects were those who underwent ocular health screening at the ophthalmology clinic of Asan medical center during the same period. The study was approved by the Institutional Review Board of Asan Medical Center and followed the tenets of the Declaration of Helsinki. On the initial visit, all participants underwent an ophthalmic examination, including a review of the medical history, measurement of best-corrected visual acuity, automated refraction, slit-lamp biomicroscopy, Goldmann applanation tonometry, gonioscopy, funduscopic photography, AL, ACD, and keratometry measurement (IOL Master; Carl Zeiss Meditec Inc.), and central corneal thickness measurement (DGH-550 instrument; DGH Technology Inc., Exton, PA, USA).

Angle assessment was conducted by one of the co-author (K.R.S.) with 23 years of experience as a glaucoma specialist. All eyes had undergone static and dynamic gonioscopy with Sussman 4-mirror gonioscope (Ocular Instruments, Bellevue, WA, USA) in a darkened room (0.5 cd/m^2^). Eyes with an occludable angle were diagnosed with PAC according to criteria set by the International Society of Geographical and Epidemiological Ophthalmology (ISGEO), including primary angle closure suspect (PACS), PAC, and PACG.^25^ Eyes with PACS were defined by their appositional contact >270 degrees between the peripheral iris and the posterior trabecular meshwork under static gonioscopic examination. The PAC group included eyes with occludable angles and had features indicating trabecular obstruction by the peripheral iris. Such features included elevated intraocular pressure (IOP), iris whorling (distortion of the radially orientated iris fibers), “glaukomflecken” lens opacities, excessive pigment deposition on the trabecular meshwork, or presence of peripheral anterior synechiae (PAS), but without the development of a glaucomatous optic disc or a change in the visual field (VF). PAC eyes showing glaucomatous optic disc changes (neuroretinal rim thinning, disc excavation, or optic disc hemorrhage attributable to glaucoma) or a glaucomatous VF change were considered to have PACG. Indentation gonioscopy was performed on all eyes to determine if the AC was due to apposition or PAS.^25^

We excluded patients younger than 50 years of age and patients with a history of acute angle closure attack, topical or systemic medications that may have affected the angle or the pupillary reflex; any ocular surgery that could affect ocular biometry results, including laser iridotomy, laser iridoplasty, vitrectomy or refractive surgery; and ophthalmic diseases that might affect the angle, including ocular trauma, uveitis, iridocorneal endothelial syndrome, or phacodonesis. In addition, eyes with ophthalmic conditions impeding measurement of AL, ACD, or keratometry, including severe media opacity, corneal disease causing irregular astigmatism, severe pterygium, or retinal disease affecting fixation were excluded. If both eyes from the same patient were eligible, one eye was selected with stratified simple random sampling using the SAS (SAS Institute Inc., Cary, NC, USA) SURVEYSELECT procedure.

Eyes with long AL or high myopia might have a different association between ACD and AL or refractive error, hence, we decided to exclude eyes longer than 25.0 mm and with myopia worse than −6.0 D.^21^^–^^24^ In addition, to address potential confounding by excessive corneal astigmatism, we have excluded eyes with corneal astigmatism of 3.0 D or more.

### Statistical Analysis

A comparison between the two groups was made with the χ^2^ test for categorical variables and independent Student's *t*-test for continuous variables after confirmation of normality. A Pearson correlation was calculated to assess the correlation between variables in all eyes.

Stepwise multiple regression models were constructed with the backward elimination method. We initially included age, gender, group (PAC vs. non-PAC), spherical equivalent (SE), K, AL, ACD, and all interaction terms that were significant in the Pearson correlation analysis. Group and gender were coded to 0 (non-PAC and male subjects) and 1 (PAC and female subjects), whereas all continuous variables were centered to zero before the analysis. Manual backward stepwise elimination was performed with a significance threshold of *P* < 0.15 and respecting the hierarchy of effects principle. That is, even if the main effect was insignificant, we removed the interaction effects first, allowing the main effects to always be included for any interaction effect in the model. We assessed the multicollinearity of the independent variables with variance inflation factors (VIFs). All statistical analysis was carried out using SAS 9.4 software.

## Results

From an initial pool of 431 non-PAC and 120 PAC subjects, 43 subjects in the non-PAC group and one subject in the PAC group were excluded according to exclusion criteria. As a result, 507 eyes of 507 subjects including 388 non-PAC eyes and 119 PAC eyes were analyzed. There was no significant difference between the PAC and non-PAC groups in terms of age (70.5 ± 7.9 years vs. 71.8 ± 8.6 years, *P* = 0.139), CCT (526.7 ± 31.1 µm vs. 535.2 ± 31.7 µm, *P* = 0.146), mean keratometry value (44.49 ± 1.44 D vs. 44.31 ± 1.48 D, *P* = 0.248), and SE (0.32 ± 1.59 D vs. 0.17 ± 1.61 D, *P* = 0.374). There were significantly more female patients in the PAC group than in the non-PAC group (79.0% vs. 51.3%, *P* < 0.001). The PAC group had significantly higher IOP (15.9 ± 5.3 mm Hg vs. 14.6 ± 3.0 mm Hg, *P* = 0.004), shorter AL (22.76 ± 0.74 mm vs. 23.44 ± 0.79 mm, *P* < 0.001) and shallower ACD (2.51 ± 0.40 mm vs. 3.08 ± 0.40 mm, *P* < 0.001) than the non-PAC group (Table 1).

**Table 1.** Baseline Characteristics of Non-PAC and PAC Eyes

	Non-PAC (*n* = 388)	PAC (*n* = 119)	*P* Value	Cohen's *d*
Age, y	71.8 ± 8.6	70.5 ± 7.9	0.139	−0.156
Sex, female	199 (51.3%)	94 (79.0%)	**<** **0** **.001**	
Laterality, right	173 (44.6%)	60 (50.4%)	0.264	
IOP, mm Hg	14.6 ± 3.0	15.9 ± 5.3	**0** **.004**	0.343
CCT, µm	535.2 ± 31.7	526.7 ± 31.1	0.146	−0.270
AL, mm	23.44 ± 0.79	22.76 ± 0.74	**<** **0** **.001**	−0.876
K, diopters	44.31 ± 1.48	44.49 ± 1.44	0.248	0.121
SE, diopters	0.17 ± 1.61	0.32 ± 1.59	0.374	0.093
ACD, mm	3.08 ± 0.40	2.51 ± 0.40	**<** **0** **.001**	−1.439

IOP, intraocular pressure; CCT, central corneal thickness; AL, axial power; K, corneal power; SE, spherical equivalent; ACD, anterior chamber depth.

*P* values with statistical significance (< 0.05) appear in boldface.

We performed a Pearson correlation analysis on the whole study population to find significant correlations among the candidate variables for the multiple regression. The PAC group showed an association with female gender, shorter AL, and shallower ACD in the correlation analysis. It revealed that female subjects had shorter AL (r = −0.38, *P* < 0.001), a steeper cornea (r = 0.22, *P* < 0.001), and a shallower ACD (r = −0.22, *P* < 0.001) than male subjects. Older age was correlated with more hyperopic refraction (r = 0.10, *P* = 0.021) and shallower ACD (r = −0.22, *P* < 0.001). Longer AL was associated with a flatter cornea (r = −0.66, *P* < 0.001), greater myopic refraction (r = −0.23, *P* < 0.001), and deeper ACD (r = 0.46, *P* < 0.001), whereas a steeper cornea was associated with a shallower ACD (r = −0.12, *P* = 0.007; Table 2^3^).

**Table 2.** A Correlation Table for the Candidate Variables for the Multiple Regression Analysis

	Group	Sex	Age	AL	K	SE
Sex	0.24^**^					
Age	−0.07	0.01				
AL	−0.35^**^	−0.38^**^	0.00			
K	0.05	0.22^**^	0.00	−0.66^**^		
SE	0.04	0.03	0.10^*^	−0.23^**^	−0.04	
ACD	−0.52^**^	−0.22^**^	−0.18^**^	0.46^**^	−0.12^**^	−0.08

AL, axial length; K, corneal power; SE, spherical equivalent; ACD, anterior chamber depth.

Values are regression coefficients.

* *P* < 0.05.

** *P* < 0.01.

**Table 3.** Final Multiple Regression Models Using Stepwise Backward Elimination

				95% CI for Beta	
Variables	Beta	t-Value	*P* Value	Lower	Upper
SE model					
PAC	−0.135	−0.59	0.556	−0.586	0.316
Age	0.022	2.67	**0** **.008**	0.006	0.038
ACD	0.178	0.82	0.412	−0.248	0.604
AL	−0.985	−7.33	**<** **0** **.001**	−1.249	−0.721
K	−0.454	−7.24	**<** **0** **.001**	−0.577	−0.331
ACD^*^ PAC	1.117	2.74	**0** **.006**	0.315	1.919
AL^*^ PAC	−0.583	−2.46	**0** **.014**	−1.050	−0.117
ACD^*^ K	0.193	1.90	0.058	−0.006	0.392
K model					
PAC	−0.448	−3.51	**<** **0** **.001**	−0.699	−0.197
Age	0.008	1.49	0.137	−0.003	0.019
ACD	0.611	4.88	**<** **0** **.001**	0.365	0.857
AL	−1.501	−23.60	**<** **0** **.001**	−1.626	−1.376
SE	−0.200	−6.91	**<** **0** **.001**	−0.257	−0.143
AL^*^ SE	0.048	1.57	0.116	−0.012	0.107
AL model					
PAC	−0.258	−4.33	**<** **0** **.001**	−0.374	−0.141
Female	−0.248	−5.44	**<** **0** **.001**	−0.337	−0.158
ACD	0.489	8.90	**<** **0** **.001**	0.381	0.597
K	−0.336	−22.61	**<** **0** **.001**	−0.365	−0.307
SE	−0.115	−8.65	**<** **0** **.001**	−0.141	−0.089
ACD^*^ K	0.046	1.56	0.119	−0.012	0.104
ACD model					
PAC	−0.412	−10.04	**<** **0** **.001**	−0.492	−0.331
Age	−0.011	−6.18	**<** **0** **.001**	−0.015	−0.008
AL	0.282	9.52	**<** **0** **.001**	0.224	0.340
K	0.073	4.82	**<** **0** **.001**	0.043	0.103
SE	0.025	2.39	**0** **.017**	0.004	0.046
AL^*^ K	−0.024	−2.04	**0** **.042**	−0.048	−0.001

SE, spherical equivalent; PAC, peripheral angle closure; ACD, anterior chamber depth; AL, axial length; K, corneal power.

Measures of fit:

SE model: R^2^ = 0.167, F = 12.50 (8, 498), *P* < 0.001.

K model: R^2^ = 0.535, F = 98.18 (6, 500), *P* < 0.001.

AL model: R^2^ = 0.675, F = 147.94 (7, 499), *P* < 0.001.

ACD model: R^2^ = 0.441, F = 66.63 (6, 500), *P* < 0.001.

* Interaction between variables:

*P* values with statistical significance (< 0.05) appear in boldface.

### Multiple Regression Analysis

Because age, gender, and biometry parameters showed complex correlations, we performed a stepwise multiple regression analysis. Using the backward stepwise elimination method, four separate multiple regression models were constructed for every biometric parameter of interest (SE, K, AL, and ACD). All four models were statistically significant (*P* < 0.001 for all), with R^2^ values of 0.167 for the SE model, 0.535 for the K model, 0.675 for the AL model, and 0.441 for the ACD model with VIFs below 2.5 for all independent variables, excluding the interaction terms. The direction of associations among the four parameters was consistent across all models (Table 3).

### Difference Between PAC and Non-PAC Eyes

PAC eyes have a significantly flatter cornea (*β* = −0.448, *P* < 0.001), shorter AL (*β* = −0.258, *P* < 0.001), and shallower ACD (*β* = −0.412, *P* < .001) than non-PAC eyes. There was no significant difference in SE between PAC and non-PAC eyes (*β* = −0.135, *P* = 0.556).

### Relationships That do not Change After Accounting for PAC (Compared to the Correlation Analysis Including the Whole Study Population)

Consistent with the correlation analysis results, female subjects had a shorter AL (AL model, *P* < 0.001) and older age was associated with greater hyperopic refraction (SE model, *P* = 0.008) and shallower ACD (ACD model, *P* < 0.001). Longer eyes had greater myopic refraction (AL model, *β* = −0.115, *P* < 0.001 and SE model, *β* = −0.985, *P* < 0.001), a flatter cornea (AL model, *β* = −0.336, *P* < 0.001 and K model, *β* = −1.501, *P* < 0.001), and deeper ACD (AL model, *β* = 0.489, *P* < 0.001 and ACD model, *β* = 0.282, *P* < 0.001).

### Relationships That Change After Accounting for PAC (Compared to the Correlation Analysis Including the Whole Study Population)

Although gender was associated with K and SE in the correlation analysis, the association was not significant in the multiple regression (both were dropped in the respective models due to a *P* > 0.15). A steeper cornea was associated with shallower ACD (*β* = −0.12, *P* = 0.007) but not with refraction (-*β* = 0.08, *P* = 0.362) in the correlation analysis, but in the multiple regression, a steeper cornea was associated with a deeper ACD (K model, *β* = 0.611, *P* < 0.001 and ACD model, *β* = 0.073, *P* < 0.001), and greater myopic refraction (K model, *β* = −0.200, *P* < 0.001 and SE model, *β* = −0.454, *P* < 0.001). In the correlation analysis, greater hyperopia was associated with shallower ACD with marginal significance (−0.08, *P* = 0.087). Although, in the multiple regression, greater hyperopic refraction was associated with deeper ACD in the ACD model (*β* = 0.025, *P* = 0.017).

The SE model required explanation because in this model, whereas the main effects of PAC and ACD were not significant (*β* = −0.135, *P* = 0.566 and *β* = 0.178, *P* = 0.412, respectively), the interaction effect of ACD*PAC was significant (*β* = 1.117, *P* = 0.006). SE may be significantly associated with ACD in PAC eyes but not in non-PAC eyes. In addition, the interaction term of AL*PAC was significant in the SE model (*β* = −0.583, *P* = 0.014), suggesting that the negative association between AL and SE (i.e. longer eyes have more myopic refraction) is even stronger in PAC eyes compared to non-PAC eyes.

### Summary of Findings From the Multiple Regression

• The association between AL and ACD was not different in PAC eyes compared with non-PAC eyes, which was consistent with the correlation analysis.
• The association between AL and SE was not different in PAC eyes compared with non-PAC eyes, which was consistent with the correlation analysis. However, the association seemed to be stronger in PAC eyes.
• A flatter cornea was associated with greater hyperopic refraction (Fig. A).
• A flatter cornea was associated with a shallower ACD (Fig. B).
• A shallower ACD was associated with greater myopic refraction in PAC eyes but not in non-PAC eyes (Fig. C).
• PAC eyes had flatter corneas than non-PAC eyes (Fig. D).

**Figure.**
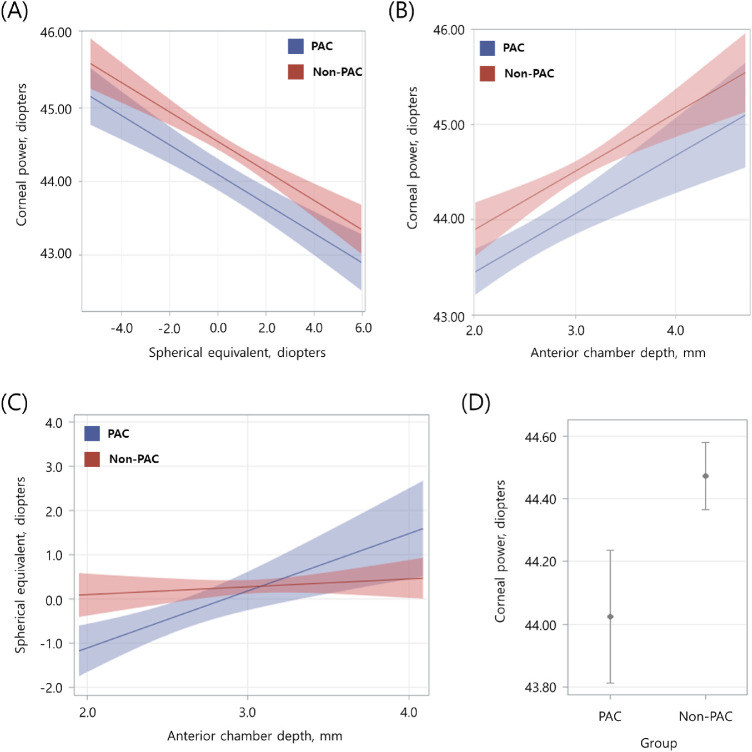
A visual depiction of the relationships between corneal power and spherical equivalent. (**A**) Corneal power and anterior chamber depth, (**B**) SE and ACD showing crossover interaction, (**C**) and calculated average corneal power (**D**) for PAC and non-PAC eyes with 95% confidence intervals. Values calculated for: **A** age = 71.5 years, AL = 23.28 mm, ACD = 2.95 mm; **B** age = 71.5 years, AL = 23.28 mm, SE = 0.20 D; **C** age = 71.5 years, AL = 23.28 mm, K = 44.35 D; **D** age = 71.5 years, AL = 23.28 mm, SE = 0.20 D, ACD = 2.95 mm. AL, axial length; K, corneal power; SE, spherical equivalent; ACD, anterior chamber depth.

## Discussion

We found that after adjusting for AL, ACD, and age, hyperopia was not associated with PAC. We also have insight into the underlying pathogenesis of PAC. First, the fundamental relationships between AL and SE and AL and ACD in PAC eyes were not different from non-PAC eyes. They may not be responsible for the development of PAC. Second, PAC eyes have a flatter cornea than non-PAC eyes, after adjusting for age, AL, ACD, and SE. That is, PAC eyes have a similar level of refractive error to non-PAC eyes despite having a flatter cornea, given their comparable AL. This implies a mechanism that compensates for the lower corneal power in PAC eyes in the rest of the refractory components, most likely in the crystalline lens.

The refractive power of the crystalline lens reportedly increases with an increase in the refractive index, with a thicker and more convex profile, and with an anterior shift. The refractive index of the crystalline lens decreases with age, but the technical difficulties associated with dynamically measuring the refractive index of the crystalline lens in vivo result in only limited evidence that suggests no change occurs during accommodation.^26^^,^^27^ The profile of the crystalline lens changes during accommodation.

Recent studies estimate that the anterior and posterior curvature radius of the lens changes approximately −0.60 mm/D and −0.20 mm/D with accommodation, causing an approximately −0.04 mm/D decrease in ACD.^28^ During accommodation, the equatorial plane position of the lens shifts anteriorly with the significant anterior shift of the anterior pole, accompanied by a much lesser or even opposite movement of the posterior pole.^28^^,^^29^ This movement may be caused by longitudinal contraction of the ciliary muscle. In monkey eyes, the ciliary muscle moved forward approximately 1.0 mm during accommodation, whereas in human eyes, the ciliary muscle has shortened about 80 µm/D until 4.0 D of stimulus and 50 µm/D from 4.0 D to 8.0 D of stimulus in response to accommodative stimuli.^30^^,^^31^ Position of the ciliary body has been shown to be the most distinct difference when subclassifying PAC eyes based on AS-OCT and UBM parameters.^32^

Based on research on the accommodative response, we can hypothesize that an accommodative response is triggered in a subgroup of eyes with insufficient corneal power to reach emmetropia but correctable with accommodation. This subsequently causes anterior chamber narrowing. With this hypothesis, our results can be explained as follows:

1) Some PAC eyes have a flatter cornea, which is insufficient to reach emmetropia without accommodation.
2) As a result, without accommodation, the ACD remains deep, but due to the insufficient refractive power of the cornea, the eye develops a hyperopic refractive error.
3) The eye accommodates to correct this hyperopic refractive error, causing the anterior surface of the lens to move forward, causing ACD to decrease.

This also can explain the finding that PAC eyes with a longer AL seem to have a flatter cornea, but a smaller relative lens position (RLP: [ACD + 1/2 lens thickness]/AL). The authors have coined this lenticular myopia.^18^

Assuming the hypothesis holds, there are still unanswered questions. First, we do not know whether the accommodative response in PAC eyes is fully reversible, as in normal eyes. A study could be designed to relieve accommodative stimuli by wearing positive glasses for a prolonged time and measuring refractive error. Or cycloplegic refraction could be measured to medically relieve accommodation; however, cycloplegia is a relative contraindication in eyes with a narrow angle. Second, we do not know if there is a long-term mechanism that adjusts the lens and the accommodation. A substantial proportion of eyes presenting with acute angle-closure (AAC) have zonular instability, but it is not clear whether those eyes represent a spectrum of PAC or are a separate entity. AAC eyes with zonular instability have longer AL, less hyperopic refraction, a larger lens vault, and a thicker lens.^33^^,^^34^ The role of vitreous zonules in accommodation and their association with PAC has recently been investigated.^35^^,^^36^

In a mathematical model of a postoperative IOL power calculation, an anterior IOL shift of 1 mm results in an approximate 1.30 D shift toward myopia.^37^ In addition, many studies have been conducted on the precise estimation of postoperative axial IOL position, as it represents the largest source of residual postoperative refractive error in modern IOL power calculations.^38^ Hence, if the crystalline lens could move forward by relaxation or elongation of the zonules, it will cause lenticular myopia, which could compensate for the flatter cornea of patients with PAC. Future research must examine if there is a mechanism that compensates for the flatter cornea over the long-term, emmetropization, or if there is a spectrum of accommodations or other distinct mechanisms.

Although we have followed strict parameter selection criteria and cross-checked our results by constructing separate models for the four parameters, results can vary depending on the model design. In addition, our study has limitations inherent to a retrospective study design.

In conclusion, PAC eyes seem to have a flatter cornea compared with non-PAC eyes, after adjusting for other parameters, but have a similar level of refractive error. A shallower ACD seems to be associated with greater myopic refraction in PAC eyes, but this relationship did not exist in non-PAC eyes.
